# Fluorescent polydopamine nanoparticles as a probe for zebrafish sensory hair cells targeted in vivo imaging

**DOI:** 10.1038/s41598-018-22828-2

**Published:** 2018-03-13

**Authors:** Gyo Eun Gu, Chul Soon Park, Hyun-Ju Cho, Tai Hwan Ha, Joonwon Bae, Oh Seok Kwon, Jeong-Soo Lee, Chang-Soo Lee

**Affiliations:** 1 Hazards Monitoring BNT Research Center, Korea Research Institute of Bioscience and Biotechnology (KRIBB), 125 Gwahak-ro, Yuseong-gu, Daejeon 34141 South Korea; 2 Disease Target Structure Research Center, Korea Research Institute of Bioscience and Biotechnology (KRIBB), 125 Gwahak-ro, Yuseong-gu, Daejeon 34141 South Korea; 3 Department of Biotechnology, University of Science & Technology (UST), 217 Gajeong-ro, Yuseong-gu, Daejeon 34113 South Korea; 4 Department of Polymer Engineering, Graduate School, Chonnam National University, Gwangju, 61186 South Korea; 5 Department of Applied Chemistry, Dongduk Women’s University, 60 Hwarang-ro 13-gil, Seongbuk-gu, Seoul 02748 South Korea

## Abstract

Fluorescent polydopamine nanoparticles (FPNPs) are prepared via the ethylenediamine (EDA)-induced degradation of as-prepared non-fluorescent polydopamine (PDA) and used for targeted bioimaging. The reductive treatment of PDA in the presence of EDA yields fluorescent precipitates, inspiring us to seek various biological approaches to preparing FPNPs with excellent optical and biocompatible properties. Moreover, we firstly found that FPNPs selectively label neuromast hair cells in the lateral line of zebrafish, their applications as a reliable fluorescent indicator to investigate the neuromast hair cells, to in turn determine the viability of hair cells, was demonstrated. FPNPs also provided a minimal toxicity enable to assay the number of functional hair cells per neuromast in live animals as development proceeds. Upon combined incubation with TO-PRO-3, a well-established hair cell marker, all hair cells that were rapidly labeled with FPNPs were observed to be also completely labeled with the TO-PRO-3, labeling hair cells in neuromasts positioned in the supraorbital, otic and occipital lateral line as well as in posterior lateral line of living zebrafish larvae. Their potential efficacy for biological applications was demonstrated by their excellent optical and biocompatible properties, offering new opportunities in cancer research, real-time monitoring of stem cell transplantation and other cell-based therapies.

## Introduction

The use of self-assembled monodisperse π-conjugated oligomers is regarded as one of the most useful strategies for the preparation of fluorescent nanoparticles^1–4^. However, their structures are unstable, and the strong hydrophobic interactions of fluorescent nanoparticles in water often decreases their fluorescence quantum yield, severely restricting their use in practical biomedical applications^5^. There is therefore still a requirement for new approaches to the development of novel fluorescent nanoparticles to overcome these limitations^6,7^.

Dopamine (DA) fulfils several important functions as a neurotransmitter in the brain, and can self-polymerize from the oxidation of catechol under alkaline conditions to generate polydopamine (PDA)^8–11^, forming a hydrophilic coating that strongly adheres to bulk materials with various shapes and surface properties^12,13^. Furthermore, the excellent physicochemical and biocompatible properties of PDA particles make this molecule suitable for investigation of various applications, including surface modification, bio-inspired hydrogels, metal deposition and drug delivery, besides being used as a coating material^14–17^. Although fluorescent polydopamine nanoparticles (FPNPs) have similar biocompatibility and fluorescence properties, few studies on PDA particles-based fluorescent nanoparticles have been reported thus far, compared to other fluorescent nanoparticles.

Here, we report a facile approach for preparation of novel FPNPs from as-prepared PDA particles, obtained directly by the ethylenediamine (EDA)-induced degradation of non-fluorescent PDA particles. The nanosized FPNP structures can be internalized in caveolae-mediated endocytosis, and in addition to adsorption onto the membrane, FPNPs may be incorporated into the cytoplasmic areas. Only a small number of previous studies have reported the cell nucleus labeling^18,19^, and, in particular, there has been no report of use of fluorescent nanoparticles as a reliable fluorescent indicator for investigating the hair cells in the lateral line of zebrafish, which could determine the viability of hair cells.

Kang *et al*. reported the fluorescence imaging of zebrafish using carbon quantum dots (C-QDs) by introducing C-QDs into embryos and larvae, enabling the observation of their distribution in the embryos and larvae of zebrafish^20^. Fent *et al*. also present the synthesis of silica nanoparticles to determine their cellular uptake and translocation in fish embryos, including evaluation of the potential toxicological risks of nanoparticles by analysis of the embryonic development. On the other hand, our labeling pattern is a stark contrast to those of other nanoparticles as imaging probes, such as carbon quantum dots and fluorescent silica nanoparticles, which display broad distribution in embryos and non-selective uptake in the chorion of the eggs^21^. The fluorescent vital dye markers to investigate the determinants of hair cell death in the lateral line of zebrafish, the well-known dyes such as FM 1-43, TO-PRO-3, and YO-PRO-1, have been typically served as indicators of hair cell viability, thereby as labeling hair cells of the lateral line *in vivo*.

Our study shows that the reductive treatment of PDA particles in the presence of EDA yields fluorescent precipitates, inspiring us to seek biological approaches to preparing FPNPs with excellent optical and biocompatible properties. Thus, we demonstrated, for the first time, that FPNPs facilitate the targeted bioimaging to investigate the neuromast hair cell in the lateral line of zebrafish, which can determine the viability of hair cells.

## Results and Discussion

**Figure 1:**
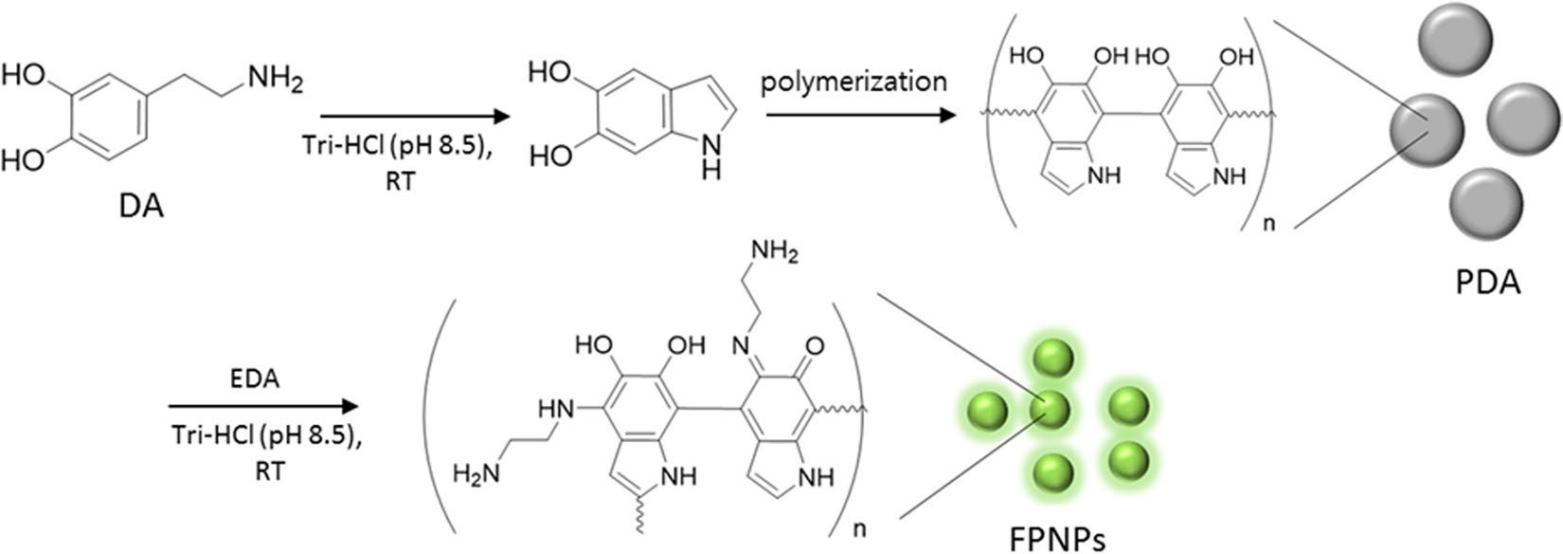
Schematic illustration of the fluorescent polydopamine nanoparticle (FPNP)s preparation process.

**Figure 2:**
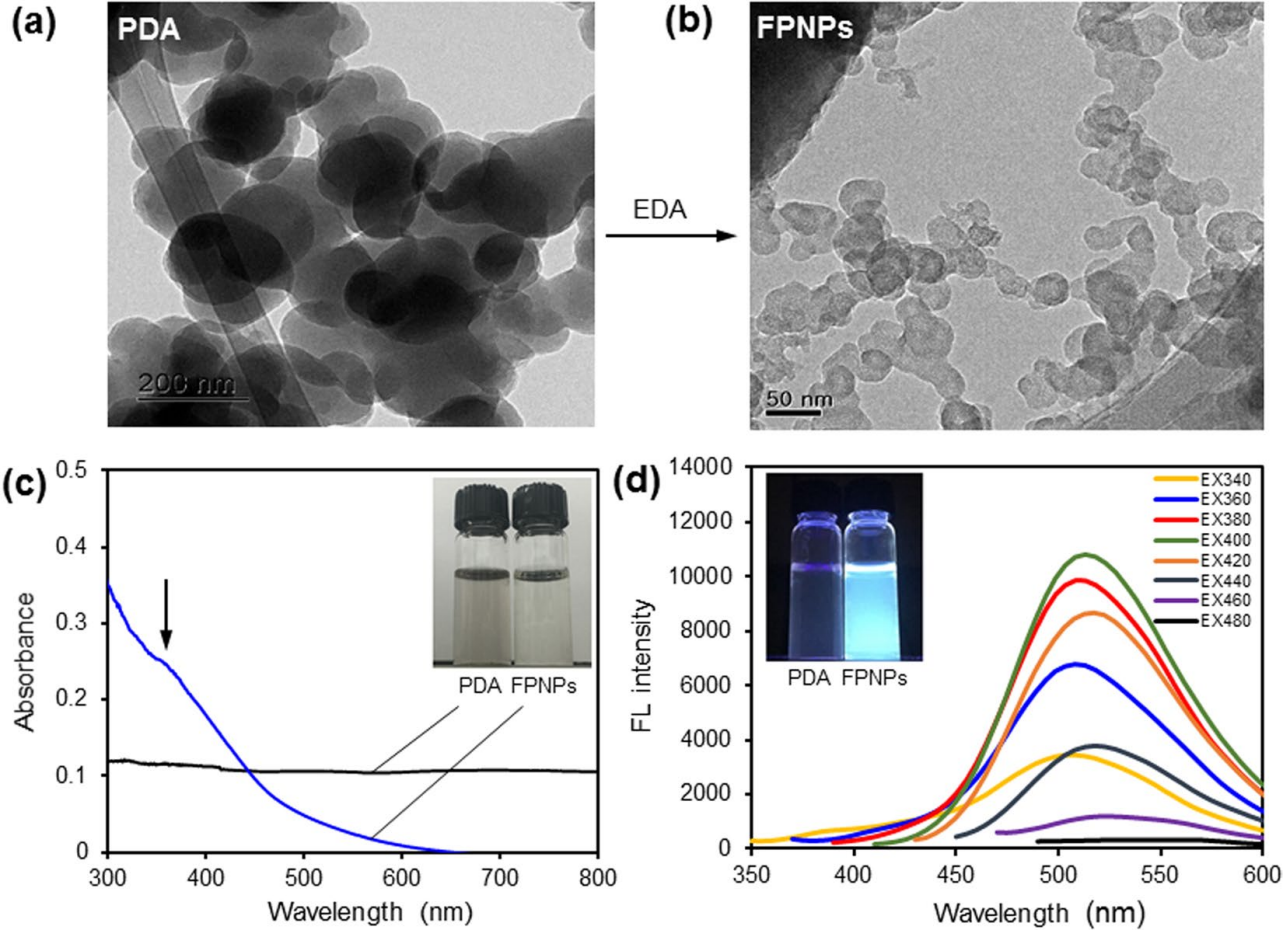
(**a** and **b**) Transmission electron microscopy (TEM) images of polydopamine (PDA particles; 1a) and fluorescent polydopamine nanoparticles (FPNPs; 1b). (**c**) Ultraviolet-visible spectroscopy (UV-vis) absorption (black) and FPNPs (blue) in water and (**d**) FL emission spectra with gradually increased excitation wavelengths from 340 nm to 480 nm after dispersion of the FPNPs in water (50 μg mL^−1^).

As shown in Fig. 1, PDA particles was initially synthesized via self-polymerization of the precursor molecule, DA, in a Tris-HCl buffer solution (pH 8.5)^22^. The resulting PDA particles was insoluble in water, and transmission electron microscopy (TEM) indicated that the morphology of the PDA particles primarily consisted of sheet-like structures 150 to 200 nm in size (Fig. 2a). As-prepared PDA particles were not water-soluble, followed by a gradual precipitation in water over time. The PDA particles was then efficiently decomposed to form FPNPs with excellent water solubility by addition to an EDA solution, thus imparting them with the suitability for subsequent functionalization with various organic, polymeric, inorganic, or biological species. The solution immediately changed to a clear phase, indicating degradation into smaller nanoparticles, and exhibited worm-like nanoparticles with diameter of tens of nanometers (Fig. 2b). Although the exact mechanism is still not known, strong π–π interactions between the aggregated PDA plates play an important role in the formation of the worm-like structure^23,24^.

Fourier transform infrared (FT-IR) spectroscopy and X-ray photoelectron spectroscopy (XPS) were used to determine the possible chemical structures of the FPNPs. The FT-IR spectrum of PDA particles exhibited two major bands, at 1620 cm^−1^ and 3420 cm^−1^ (Fig. S1b), which originated from the C=C and O–H stretching vibrations, respectively. This result is consistent with previously reported FT-IR results for PDA particles^25–28^.

These two major bands were also observed in the FPNP spectrum (Fig. S1c), suggesting that PDA particles functional groups are preserved in FPNPs. Additionally, the increased peak centered at 2960 cm^−1^ was attributed to the sp3 C-H stretch by reaction with EDA. C, N, and O were found in the PDA particles and FPNPs from the XPS spectrum, as shown in Fig. S2. The PDA particles and FPNPs showed no difference in the types of elements, except for an increase in the N element content by addition of EDA. The XPS results indicate that the surface of the FPNPs was functionalized by N-containing groups by the reaction between PDA particles and EDA.

In the ultraviolet-visible (UV-vis) spectra, FPNPs typically show obvious optical absorption in the UV region, with a tail extending to the visible range, and its shoulder was observed at 350–400 nm; however, it should be noted that PDA particles alone are non-emissive in the UV/vis range (Fig. 2c). These results support the hypothesis that the fluorescence of FPNPs arises from incorporation of small fluorescent molecules formed during degradation by a reaction between PDA particles and EDA, rather than a modification of the existing PDA particles. As reported in previous studies, the presence of an extended π–π stacking interaction between the oligomeric units in PDA particles^29^, results from a considerable decrease in the particle size of FPNPs rather than that of PDA particles, suggesting that FPNPs had a lower degree of π–π interaction. Moreover, the presence of additional amine groups in FPNPs can effectively prevent the conjugated backbone from π–π stacking. Because the π–π stacking interaction can induce fluorescence quenching^30–32^, strong fluorescence induced by the FPNPs was observed. The fluorescence intensity of FPNPs varied when the excitation wavelength was changed from 340 to 480 nm, reaching its maximum when they were excited by 400 nm light.

However, the FPNP emission peaks appeared to be excitation-independent, without a noticeable shift toward longer wavelengths with increasing of excitation wavelength. This can be explained by a single transition mode and the narrow size distribution of the FPNPs^33–36^, implying that amino groups could be an effective candidate for controlled surface passivation of FPNPs and consequent determination of the luminescence character, making it excitation independent. Since the photostability of the fluorescent probe is significant for fluorescent detection *in vitro* and *in vivo*, the change in fluorescent intensity was evaluated after continuous irradiation at 490 nm by a 150 W cm^−2^ state xenon lamp in a Tris-HCl buffer (10 mM, pH 8.5) for 3,000 seconds. The changes in fluorescence intensity of the FPNPs solution at λ_em_ = 525 nm are negligible, indicating excellent photostability under the detection condition (Fig. S4).

Furthermore, the fluorescence decay kinetics of FPNPs in water exhibited multiexpotential fluorescence decay, with average excited-state lifetimes of 4.73 ns for emission at 518 nm when was excited at 375 nm, exhibiting excellent stability (Fig. S5). In contrast to typical organic fluorophores, such as Cy5 (*τ*_av_ = 1.5), Nile Red (*τ*_av_ = 3.6) and fluorescein (*τ*_av_ = 4.0), FPNPs exhibited a longer experimental fluorescence decay time in water. This allows temporal discrimination of the signal from cellular auto-fluorescence and the scattered excitation light by time-gated measurements, thereby enhancing the sensitivity^37^.

**Figure 3:**
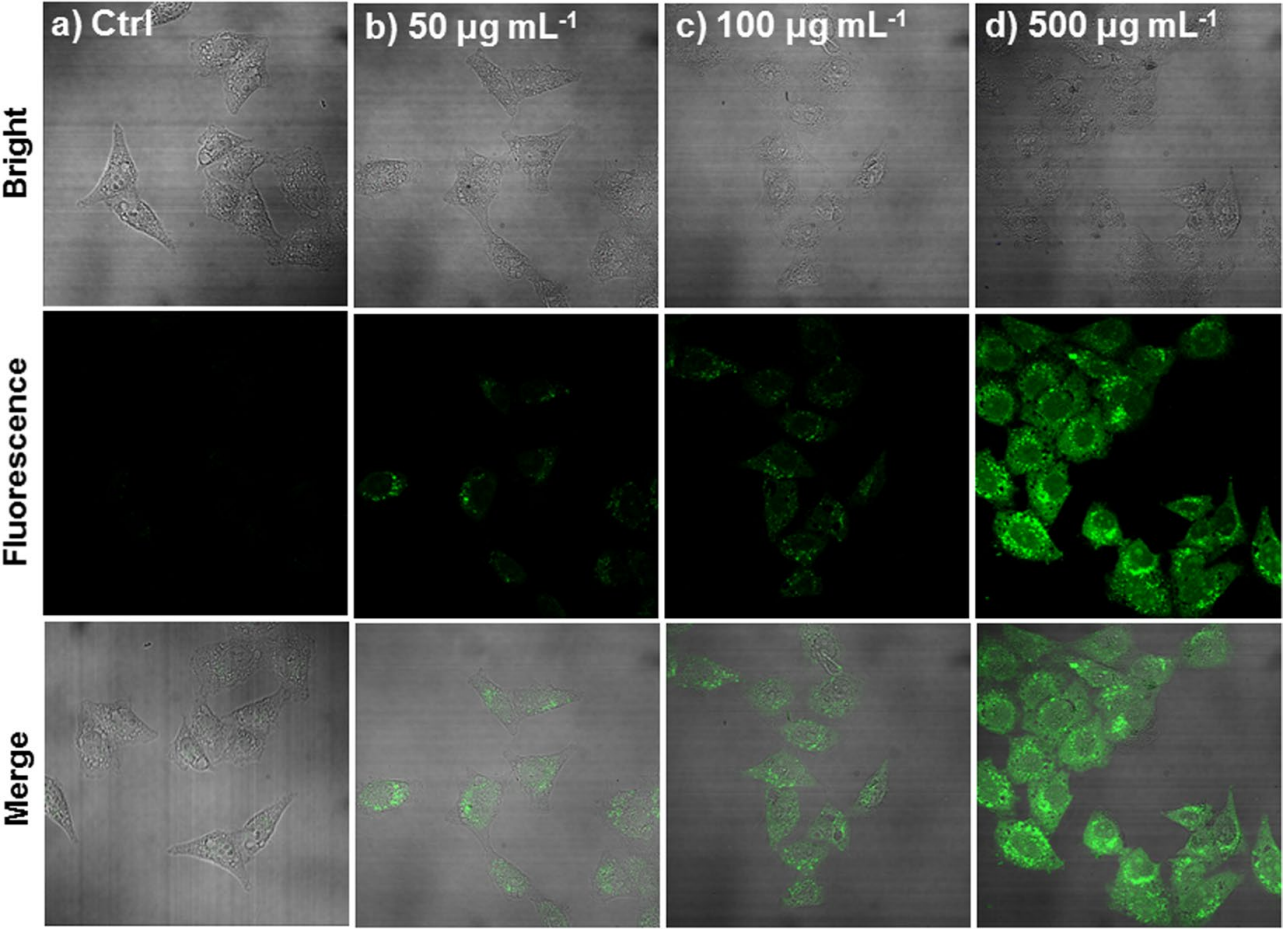
Confocal fluorescence images of living HeLa cells incubated with FPNPs of 0 μg mL^−1^, (**b**) 50 μg mL^−1^, (**c**) 100 μg mL^−1^ and (**d**) 500 μg mL^−1^. Incubation was performed at 37 °C under a humidified atmosphere containing 5% CO_2_. The fluorescence was determined at 488 nm with excitation at 405 nm.

To establish the potential efficacy for biological applications, based on the excellent fluorescence stability and biocompatible properties of FPNPs, we examined fluorescence images of FPNPs in living cells using a confocal microscope. CCK-8 assays were conducted, and the results showed that >95% HeLa cells survived after 1 h incubation with FPNPs (0–500 μg mL^−1^). The cell viability remained at approximately 90% after 24 h, demonstrating that FPNPs are not cytotoxic toward cultured cell lines (Fig. S6). Investigation of the cell permeability of FPNPs showed green emission from the green channel (450–555 nm), establishing the efficacy of FPNPs for uptake and accumulation in cells. A marked difference in fluorescence intensity was observed with differing FPNPs concentration (0, 50, 100 and 500 μg mL^−1^), mainly in the cytoplasm (Fig. 3). Generally, fluorescent nanoparticles have typically been done with cells *in vitro*, in contrast, *in vivo* interactions between cells and them are commonly hard to characterize since they are difficult to image and often appear at unexpected locations. Therefore, a novel approach is needed to increase the efficiency of these interactions, allowing the use of FPNPs to identify where an interaction occurs.

**Figure 4:**
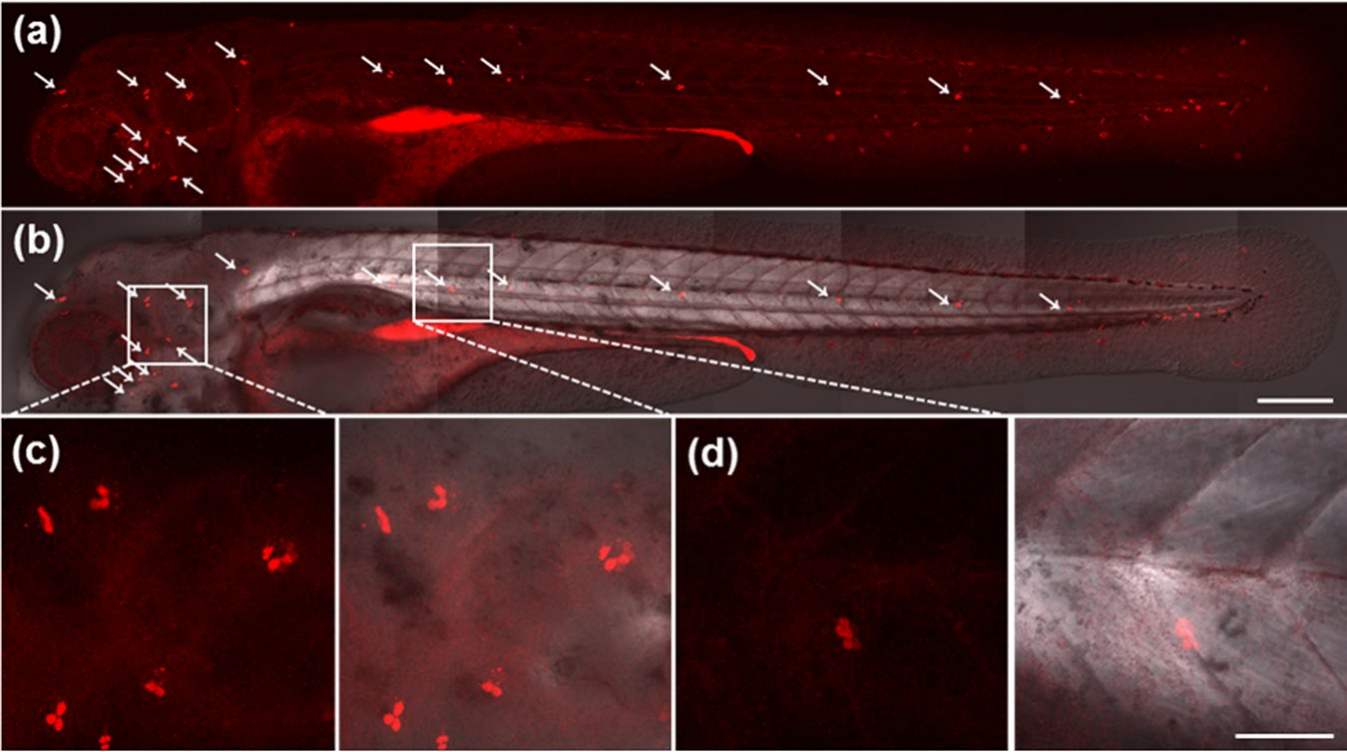
Sensory hair cells in the lateral line of whole zebrafish larvae at 4 dpf. White arrows (**a** and **b**) show the lateral line hair cells in a zebrafish larva incubated with FPNPs (2 mg mL^−1^) from 2 to 4 dpf. (**a**) Fluorescence image, (**b**) Merged image with DIC. Enlarged images of (**c**) cranial neuromasts and (**d**) trunk neuromasts. The fluorescence was determined at 543 nm with excitation at 405 nm. Scale bars: 200 μm (**a,b**), 50 μm (**c,d**).

Zebrafish larvae were chosen as a model for *in vivo* optical imaging by incubation with FPNPs, enabling the visualization of embryo development. The embryos were incubated in a 2 mg mL^−1^ FPNP solution, and the fluorescence images were observed on the hair cells of the lateral line at 4 days post-fertilization (dpf) (Fig. 4). As an attracting animal model for auditory studies, zebrafish is not only highly comparable with humans, but also greatly accessible to the hearing organ^38^. Interestingly, FPNPs appeared to selectively label the entire lateral line consisting of stereotypical patterns and numbers of neuromasts along the body of the developing zebrafish larvae (Fig. 4)^20^, with a minimal toxicity on the survival (data not shown). This labeling pattern is a stark contrast to those of other nanoparticles as imaging probes, such as carbon quantum dots and fluorescent silica nanoparticles, that display broad distribution in embryos and non-selective uptake in the chorion of the eggs^21,39^.

**Figure 5:**
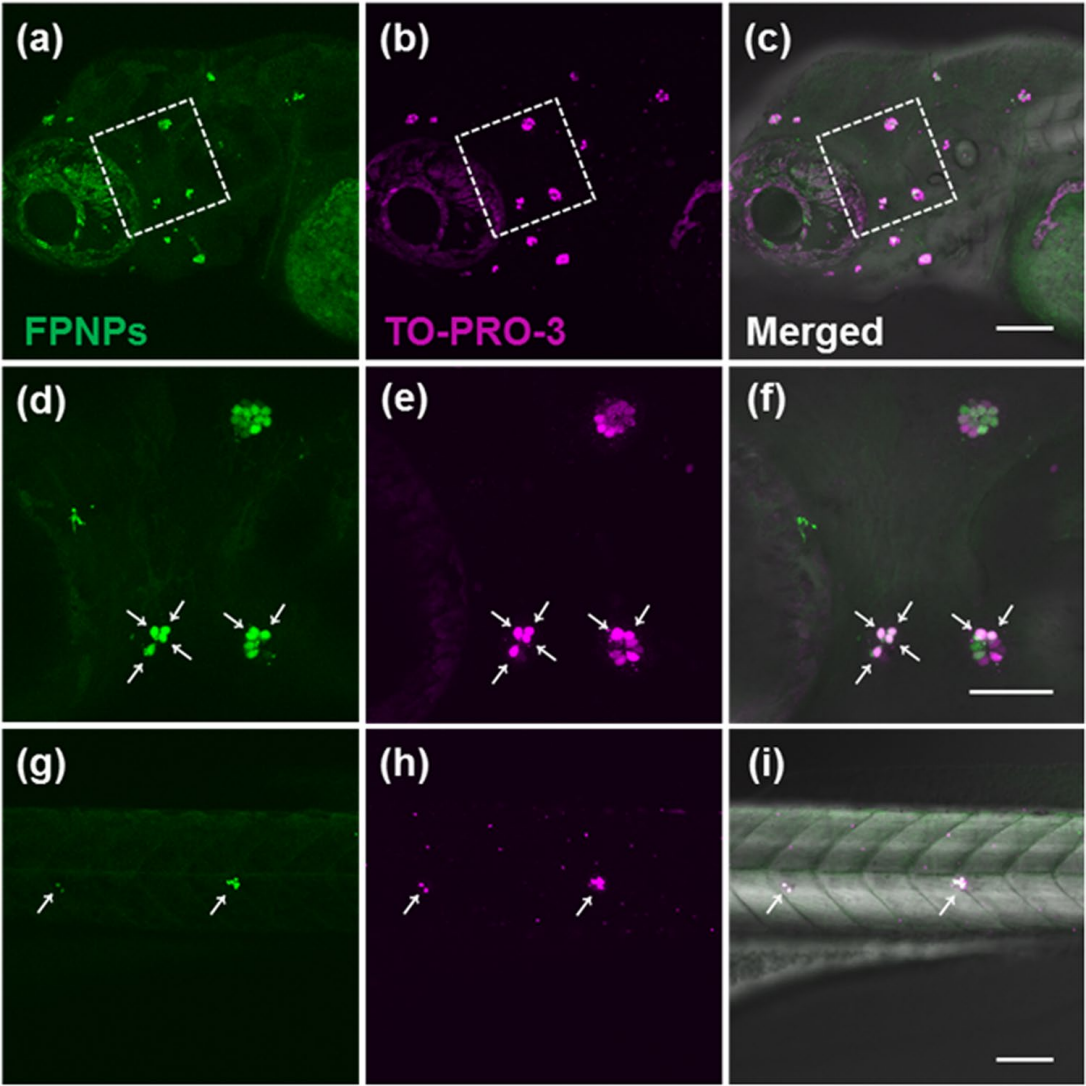
Live hair cells are double labelled with FPNPs (2 mg mL^−1^) and TO-PRO-3 (2 μM) in zebrafish. The panels (**a**,**d** and **g**) show the cells stained with FPNPs (excitation wavelength at 405 nm, emission wavelength at 488 nm, respectively). The panels (**b**,**e** and **h**) show the fluorescence images by TO-PRO-3 (excitation wavelength at 635 nm, emission wavelength at 647 nm, respectively). The panels (**c**,**f** and **i**) show the overlay images of (a) + (b), (d) + (e), and (g) + (h), respectively. White arrows (**d**,**e**, **f**,**g**,**h** and **i**) indicate the co-stained hair cells. The enlarged images of the white boxed areas in (**a**,**b** and **c**) correspond to (**d**),(**e**), and (**f**), respectively. Scale bars: 50 μm (**a**–**f**), 100 μm (**a**–**c** and **g**–**i**).

To further confirm the identity of the FPNP-labeled cells and assess effects on the neuromast development, we incubated zebrafish larvae at 4 dpf using a combination of the TO-PRO-3 dye and FPNPs. The TO-PRO-3, a cyanine dye that binds to DNA upon entry into the cell and becomes fluorescent, selectively labels active hair cells when the cells are exposed to the dye in diverse animals, including zebrafish^40^. Labeling hair cells of the lateral line *in vivo* with TO-PRO-3 have been used to record hair cell survival^40^. As expected, TO-PRO-3 labeled hair cells in neuromasts positioned in the supraorbital, otic and occipital lateral line as well as in posterior lateral line of living zebrafish larvae (Fig. 5b,e,h)^41^.

Upon combined incubation, all hair cells that were rapidly labeled with FPNPs were observed to be also completely labeled with the TO-PRO-3 dye (Fig. 5). To the best of our knowledge, FPNP is the first reported fluorescent nanoparticle that can specifically label neuromasts with a minimal toxicity, which will enable to assay the number of functional hair cells per neuromast in live animals as development proceeds. Since zebrafish lateral line hair cells and human hair cells in the ear are anatomically and functionally very similar^42^, taking advantages of the zebrafish lateral line as a model system and the high stability, selectivity, and modifiability of FPNPs warrants significant contribution to our understanding of the molecular control of hair cell development and regeneration in real time at the single cell level upon damages such as aminoglycoside antibiotics^43^.

In addition, we also investigated targeting effects by labeling using FPNPs prepared with different aliphatic diamines (1,4-butanediamine; 1,4-BDA, 1,8-octanediamine; 1,8-ODA) used as passivation agents. Although their optical and physical properties were similar to those of EDA-based FPNPs (Figs S9~S11), fluorescence signals from selective cell uptake were not observed in zebrafish lateral line hair cells with treatment of either FPNPs (Fig. S7). Instead, co-incubation with TO-PRO-3, as a well-known labelling dye for hair cells of the lateral line *in vivo*, exhibited cytotoxicity on neuromast hair cells while leaving presumptive fragmented DNA/cell debris, which are the hallmarks of cells undergoing cell death (Fig. S8), affirming divergent effects of distinct FPNPs on cytotoxicity despite their similar chemical composition as well as the importance of *in vivo* validation before applications.

Currently, the exact mechanism for the selective entry of FPNPs into hair cells is not clear: it may occur by endocytosis, a major means for a cell to incorporate large-sized polar molecules inside generally, or by passing through large non-selective cationic channels such as TRPV1 (transient receptor potential cation channel subfamily V member 1) or a P2X_2_ purinergic receptor similar to fluorescent dyes that specifically label sensory neurons such as styryl pyridinium dyes (FM1-43, DASPEI) and cyanine dyes (TO-PRO-3, YO-PRO-1)^40,44^. The second possibility is supported by the previous reports that (1) zebrafish hair cells express a large collection of non-selective cationic channels including P2X_7_ and TRPV1^45–48^; (2) HeLa cells, the cell line used in our experiments for FPNP entry *in vitro* (Fig. 3), also express a truncated P2X_7_ receptor^49^; (3) nanometer-sized dyes can pass through the P2X_7_ receptors^50^. However, further studies are required to determine the actual means of labeling specific cell types by FPNP and to understand its underlying molecular mechanisms in detail.

## Conclusions

In conclusion, the highly water-soluble FPNPs were prepared using a simple method based on PDA particles degradation by EDA. Their potential efficacy for biological applications was demonstrated by their excellent optical and biocompatible properties, which offer new opportunities in cancer research, real-time monitoring of stem cell transplantation and other cell-based therapies. Moreover, we found for the first time that FPNPs selectively label neuromast hair cells in the lateral line of zebrafish. Their applications as a reliable fluorescent indicator to investigate the neuromast hair cells, to in turn determine the viability of hair cells, was demonstrated, comparing with TO-PRO-3, a fluorescent dye typically served as indicator of hair cell viability. Deeper understanding on mechanisms of development and de-/re-generation of neuromasts upon damage, and novel therapeutic approaches, assisted by successful application of FPNPs, are forthcoming.

## Materials and Methods

This study was approved by Korea Research Institute of Bioscience & Biotechnology (KRIBB). All experiments were performed in accordance with the relevant guidelines and regulations.

### Materials and characterization

Dopamine hydrochloride and ethylenediamine (EDA; 99.5% purity) were purchased from Sigma-Aldrich. All other agents and solvents were purchased from commercial sources and used directly without further purification. A TO-PRO-3 Iodide (642/661) 1 mM solution in dimethyl sulfoxide (DMSO) was obtained from Thermo Fisher. UV/Vis and fluorescence spectra were obtained using a Beckman Coulter DU800 spectrophotometer and a Scinco Fluoromate FS-2 spectrometer, respectively. The TEM images were taken with a FEI Tecnai G2 F30 S-TWIN Transmission Electron Microscope (300 kV). FTIR spectra were recorded using an Alpha-P Bruker optics Fourier transform infrared spectrometer. The XPS analysis was conducted using a PHI VersaProbe XPS microprobe with a monochromatic X-ray source Al Kα excitation (1486.6 eV). The fluorescence lifetime was measured using a Horiba FM-4P time-corrected single photon counting system. The fluorescence decay curves were recorded using excitation at 375 nm and an emission wavelength of 518 nm.

### Preparation of FPNPs

Dopamine hydrochloride (200 mg, 1.31 mmol) was dissolved in a Tris-HCl buffer (40 mL, 100 mM, pH 8.5) and stirred at room temperature for 8 h at 500 rpm, followed by collection of PDA particles through purification. EDA was then added to the solution followed by stirring for another 20 h at room temperature. The obtained solution changed from black to reddish brown. The solution was purified using a dialysis tube (molecular weight cutoff, 100–500 Da) against deionized (DI) water for 3 days at 70 rpm. Finally, the purified products (FPNPs) were freeze-dried and characterized using a number of techniques.

### Cytotoxicity test (CCK-8 assays)

HeLa cells were plated in flat-bottomed, 96-well plates at a density of 5 × 10^3^ cells/well in 200 μL of Dulbecco’s modified Eagle’s medium (DMEM) (GIBCO, 11885) supplemented with 10% (v/v) FBS and 1% penicillin/streptomycin in a humidified incubator at 5% CO_2_ in air at 37 °C. Following incubation for 24 h, FPNPs in distilled water were added to the above cellular sample plates. After incubation for 30 min, 10 μL of Cell Counting Kit-8 (CCK-8) solution (Dojindo, Japan) was added to each plate well, and the cells were further incubated for 30 min. The absorbance at 450 nm was measured with a microplate reader (SpectraMax M2; Molecular Devices).

### Preparation and imaging of zebrafish embryos

#### Zebrafish

Zebrafish (*Danio rerio*) AB (wild type) embryos were spawned by conventional mating of the adult zebrafish AB male/female pairs. Collected embryos were maintained in 100 embryos per 100 mm^2^ petri dish with E3 Egg water (5 mM NaCl, 0.17 mM KCl, 0.33 mM CaCl_2_, 0.33 mM MgSO_4_) at 28.5 °C incubator. In order to obtain transparent zebrafish for confocal microscopy, embryos were incubated with 1 × PTU (0.003% 1-phenyl 2-thiourea, Sigma-Aldrich)-E3 egg water after 6 hours post-fertilization (hpf). All animal procedures are approved by KRIBB-IACUC with the approval number KRIBB-AEC-17122.

#### FPNPs and TO-PRO-3 staining

Zebrafish larvae were transferred into 6 well plates with 1 × PTU E3-Egg water each containing EDA/FPNPs, 1,4-BDA/FPNPs, or 1,8-ODA/FPNPs of 2 mg mL^−1^. Approximately 20 embryos (dechorionated) per each well of 6-well plate were incubated from 2 to 4 dpf or briefly treated for 4 hours at 4 dpf for the experiments. To label hair cells of lateral line, 2 μM of TO-PRO-3 Iodide (ThermoFisher Scientific, Cat. # T3605) was used for 1 hour followed by three time washing with 1 × PTU E3-Egg water. As a control, 1 × PTU-E3 Egg water containing 0.2% DMSO was used.

#### Imaging analysis

To image zebrafish larvae alive, larvae were anesthetized with Tricaine (3-amino benzoic acidethylester, Sigma) solution and embedded with 2.5% methyl cellulose in E3 Egg water. Imaging of zebrafish larvae was performed using Olympus FV1000 confocal microscopy. The labeled larvae were imaged at excitation-emission spectra of 405/488 or 405/543 for FPNPs, and 635/647 for TO-PRO-3 Iodide.

## Electronic supplementary material

Supplementary Information
