# Supplementary material for: Fluorescent polydopamine nanoparticles as a probe for zebrafish sensory hair cells targeted in vivo imaging
Source: Sci Rep. 2018 Mar 13;8:4393. doi: 10.1038/s41598-018-22828-2 (PMC5849738; doi:10.1038/s41598-018-22828-2)
Supplement: Supplementary file 1 — Supplementary Information [file 41598_2018_22828_MOESM1_ESM.docx]

**Supporting Information for**

**Fluorescent polydopamine nanoparticles as a probe for zebrafish sensory hair cells targeted in vivo imaging**

Gyo Eun Gu^1,§^, Chul Soon Park^1,4,§^, Hyun-Ju Cho^2,3,§^, Tai Hwan Ha^1,3^, Joonwon Bae^5^, Oh Seok Kwon^1,3^, Jeong-Soo Lee^2,3,^* and Chang-Soo Lee^1,3,^*

^1^Hazards Monitoring BNT Research Center, Korea Research Institute of Bioscience and Biotechnology (KRIBB), 125 Gwahak-ro, Yuseong-gu, Daejeon 34141, South Korea. ^2^Disease Target Structure Research Center, Korea Research Institute of Bioscience and Biotechnology (KRIBB), 125 Gwahak-ro, Yuseong-gu, Daejeon 34141, South Korea. ^3^Department of Biotechnology, University of Science & Technology (UST), 217 Gajeong-ro, Yuseong-gu, Daejeon 34113, South Korea. ^4^Department of Polymer Engineering, Chonnam National University, Gwangju 61186, South Korea. ^5^Department of Applied Chemistry, Dongduk Women’s University, Seoul 02748, South Korea.

^§^ These authors contributed equally to this work.

Correspondence and requests for materials should be addressed to J.S.L. (email: jeongsoo@kribb.re.kr) or C.-S.L. (email: [cslee@kribb.re.kr](mailto:cslee@kribb.re.kr))

***Fourier Transform Infrared (FT-IR) and X-ray photoelectron spectroscopy (XPS) analysis***

FT-IR spectroscopy and XPS were used to determine the possible chemical structures of the FPNPs. The FT-IR spectrum of PDA exhibited two major bands, at 1,620 and 3,420 cm^-1^ (Figure S1b), which originated from the C=C and O–H stretching vibrations, respectively. These two major bands were observed in the FPNP spectrum (Figure S1c), suggesting that the PDA functional groups are preserved in FPNPs. The increased peak centered at 2,960 cm^-1^ was attributed to the sp3 C-H stretch by reaction with EDA. The XPS analysis showed the composition of as-synthesized PDA and FPNPs. C, N, and O were found in the PDA and FPNPs from the XPS spectrum, as shown in Figure S2. The C1s peak was resolved into three components centered at 284.5, 285.8, and 288.2 eV, which represent C-C/C-H, C-OH/C-N and C=O bonds, respectively. Compared to PDA, a relative increase in the C-C/C-H bonds and a decrease in the C-OH/C-N bond in FPNPs was observed after the addition of EDA (Figure S3a, S3b). The O1s peaks can be resolved into two components centered at 531.5 eV and 532.7 eV, representing the presence of the C=O and C-OH group originating from the quinone and catechol groups in both PDA and FPNPs. An increase in the C=O bond from the quinone group was observed during FPNP preparation. The N1s spectra showed the presence of amines (398.6 eV), nitriles (399.7 eV) and oximes (401.6 eV) in both PDA and FPNPs; however, N1s from the appearance of the amide bond (400.7 eV) and the increase in the amine group (398.6 eV) were observed after FPNP preparation (Figure S3e, S3f). The XPS results indicate that the surface of the FPNPs is functionalized by multiple C, O and N-containing groups by the reaction between PDA and EDA.


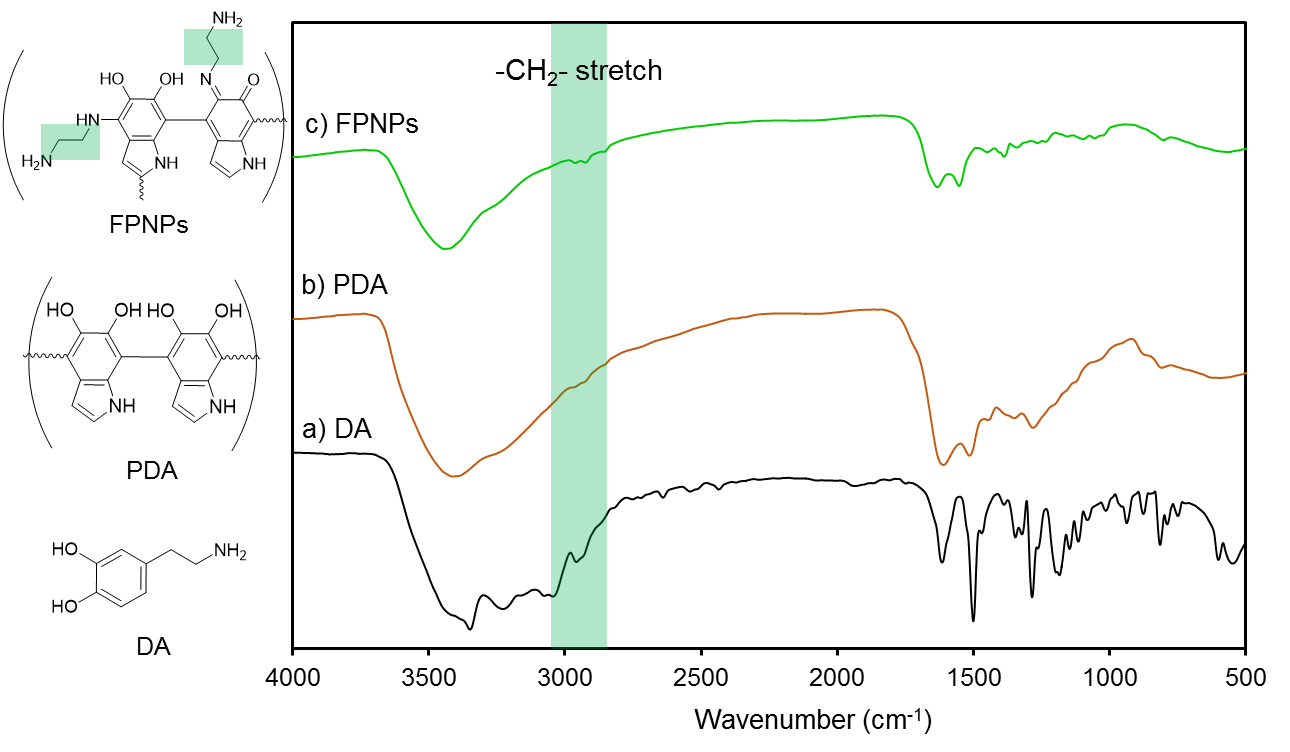


**Figure S1.** Fourier transform infrared (FT-IR) spectra of dopamine (a), polydopamine (PDA) (b) and fluorescent polydopamine nanoparticles (FPNPs) (c).


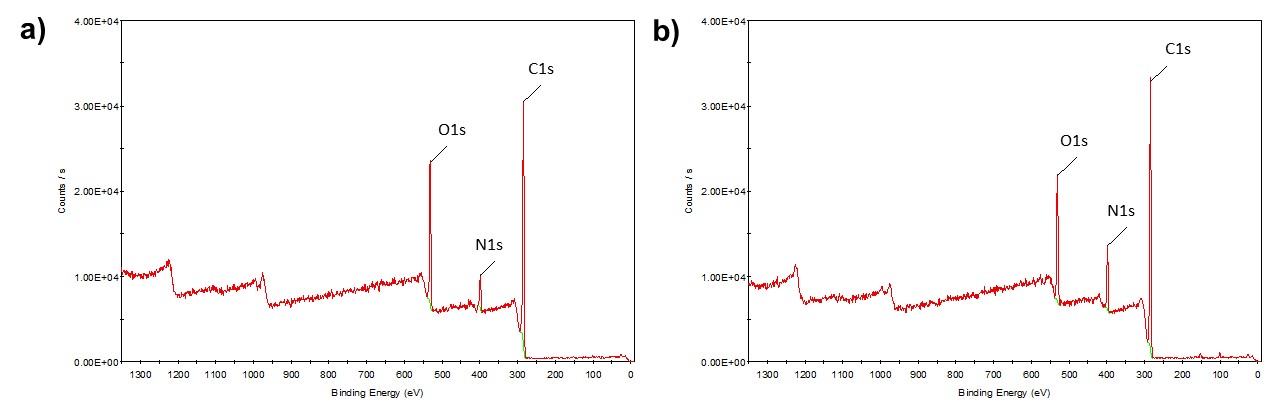


**Figure S2.** X-ray photoelectron spectroscopy (XPS) spectra survey of PDA (a) and FPNPs (b). The survey scans show that the nitrogen peak of FPNPs is relatively higher than that of PDA. Also, in the element ratio, FPNPs (C: 76.32%, O: 13.11% and N: 10.57%) increased the ratio of nitrogen to PDA (C: 76.84%, O: 16.64% and N: 6.52%), and the ratio of oxygen decreased.


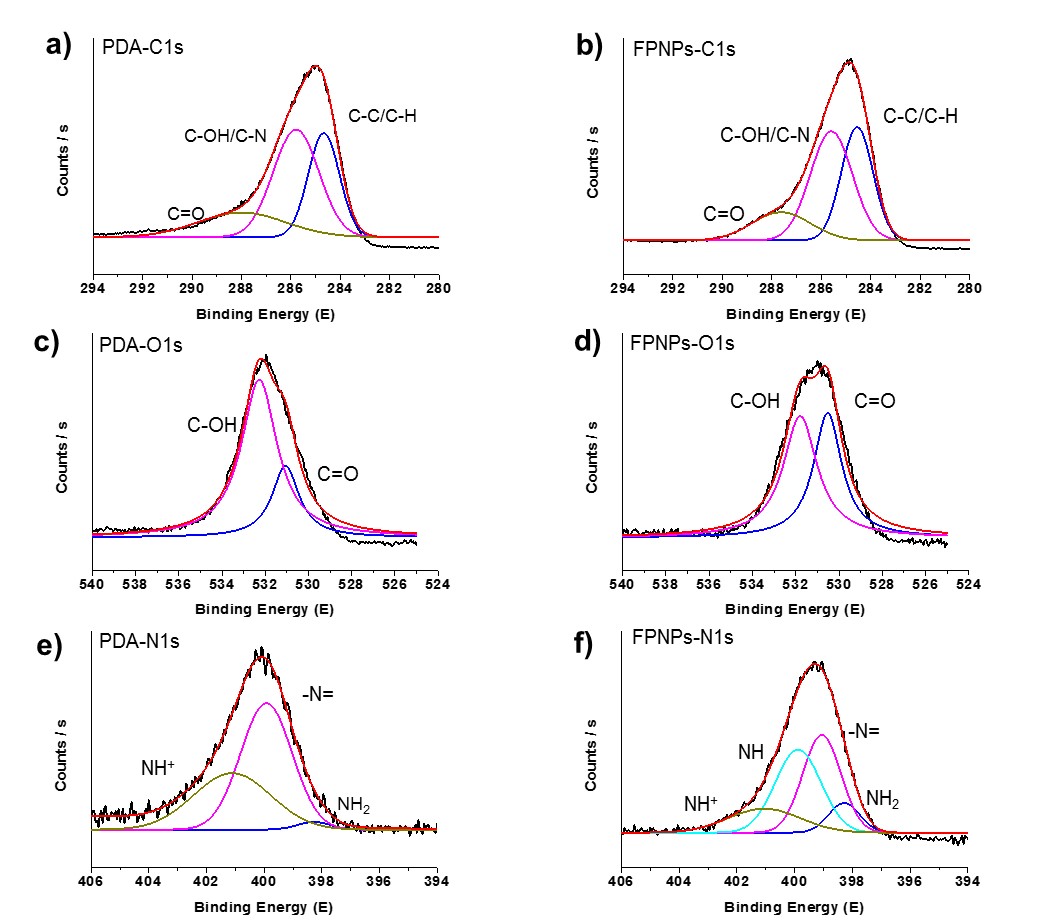


**Figure S3.** XPS C1s, O1s, and N1s spectra of PDA (a, c, e) and FPNPs (b, d, f).
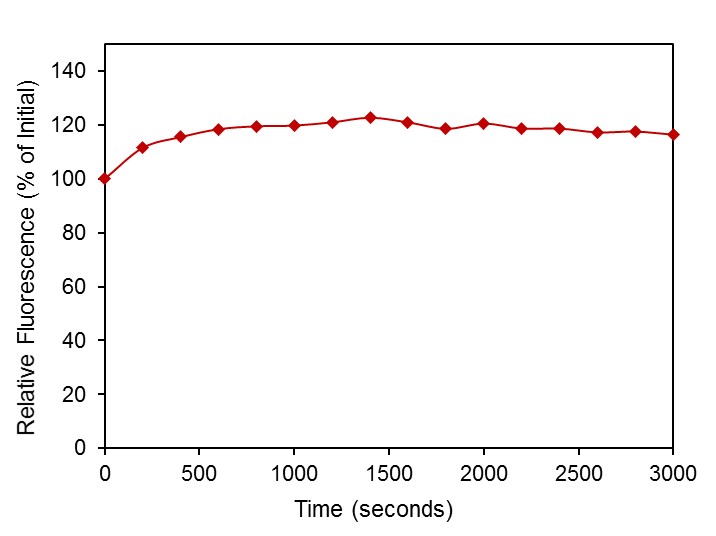


**Figure S4.** Changes in fluorescent intensity (at λ_em_ = 525 nm) of FPNPs after continuous illumination at 400 nm by a 150 W xenon lamp in a Tris-HCl buffer (10 mM, pH 7.4) for 3,000 s.


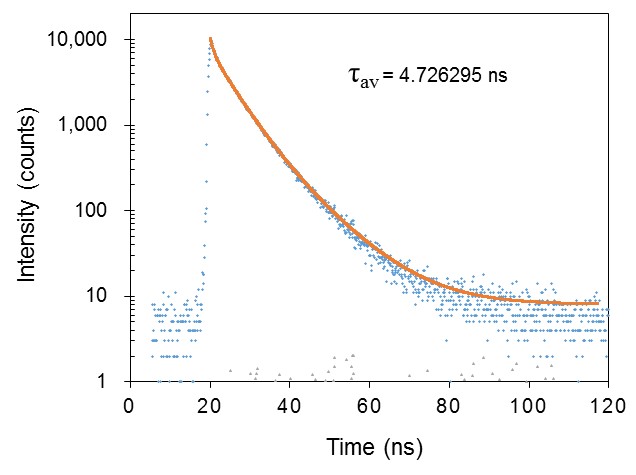


**Figure S5.** Experimental fluorescence decay curve of FPNPs in water. The orange line corresponds to double exponential fits to the data. τ_av_ is the average fluorescence lifetime calculated from the multiexponential decay fit.

***Cytotoxicity test (CCK-8 assays)***

HeLa cells were plated in flat-bottomed, 96-well plates at a density of 5,000 cells/well in 200 μL of Dulbecco’s modified Eagle’s medium (DMEM) (GIBCO, 11885) supplemented with 10% (v/v) FBS and 1% penicillin/streptomycin in a humidified incubator at 5% CO_2_ in air at 37 °C. Following incubation for 24 h, FPNPs in distilled water were added to the above cellular sample plates. After incubation for 30 min, 10 μL of Cell Counting Kit-8 (CCK-8) solution (Dojindo, Japan) was added to each plate well, and the cells were further incubated for 30 min. The absorbance at 450 nm was measured with a microplate reader (SpectraMax M2; Molecular Devices).


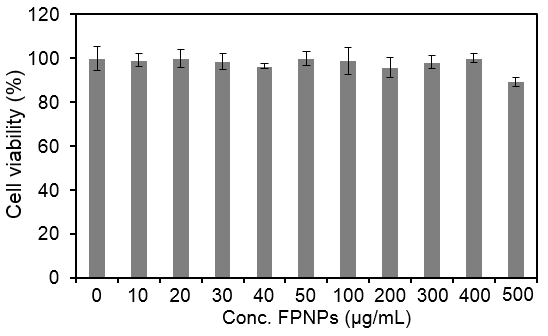


**Figure S6.** Viability of HeLa cells in the presence of FPNPs as measured by using CCK-8 kit. The cells were incubated with 0–500 μg mL^-1^ FPNPs for 24 h.


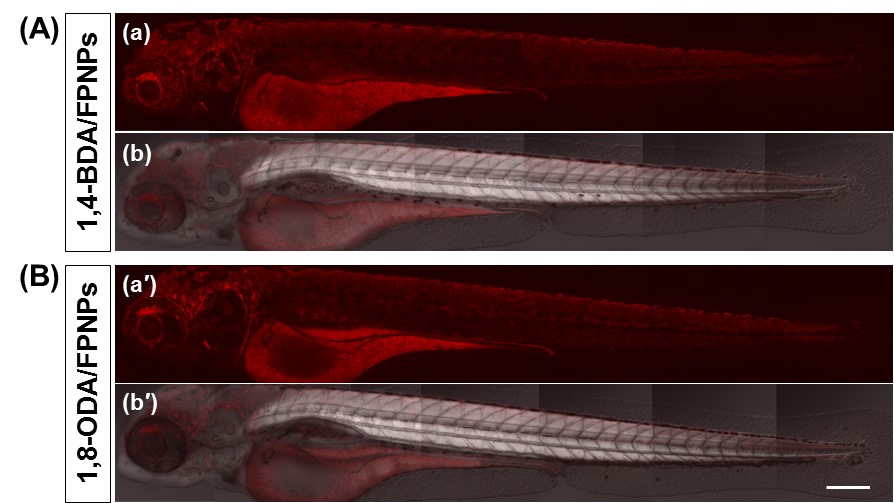


**Figure S7.** Live staining of hair cells with (A) 1,4-BDA/FPNPs and (B) 1,8-ODA/FPNPs (2 mg mL^-1^) at 4 dpf. (a and a′) excitation at 405 nm, emission at 543 nm, (b and b′) merged images with DIC, scale bar: 200 μm.


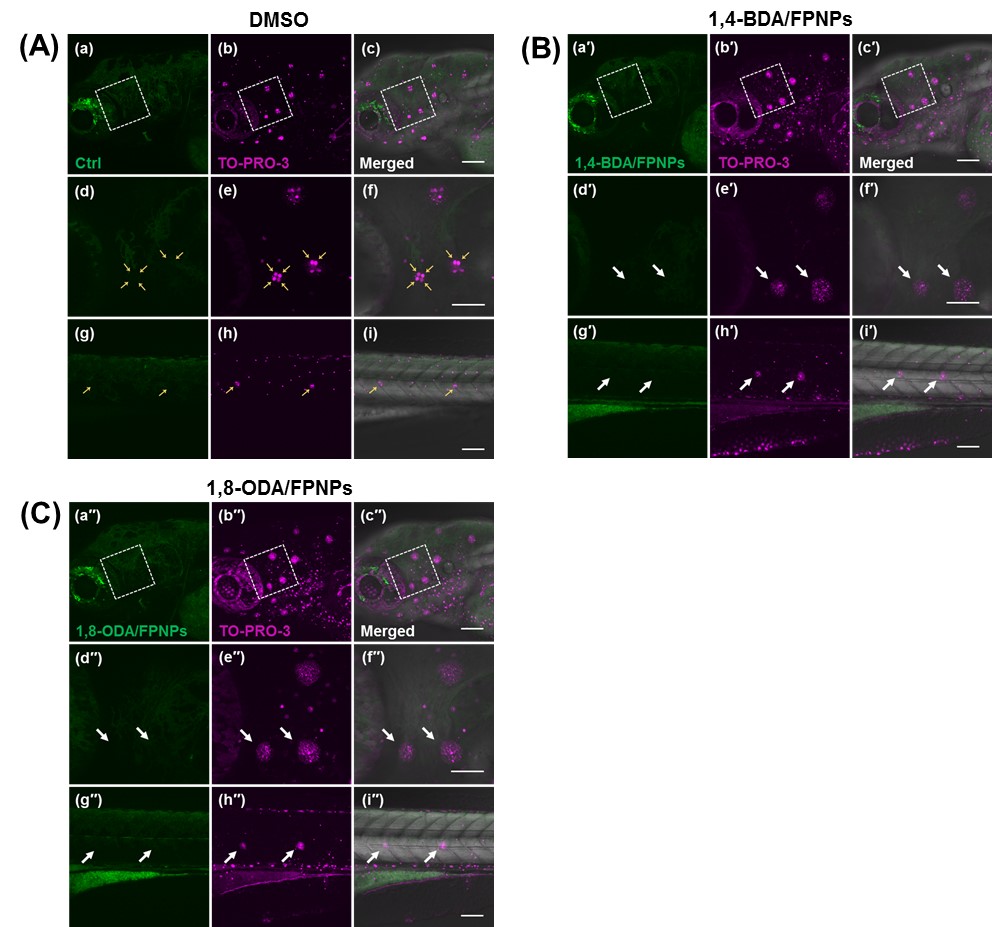


**Figure S8.** 4 Live hair cells are double labelled with FPNPs (2 mg mL^-1^) and To-PRO^TM^-3 (2 μM) in zebrafish at 4 dpf. (A) DMSO control; (B) 1,4-BDA/FPNPs; (C) 1,8-ODA/FPNPs. The panels a, a', a'', d, d', d'' and g, g', g'' show the cell targeting images of DMSO control, 1,4-BDA/FPNPs and 1,8-ODA/FPNPs (excitation at 405 nm, emission at 488 nm), respectively. The panels (b, b', b''), (e, e', e'') and (h, h', h'') show the To-PRO^TM^-3 fluorescence staining (excitation at 635 nm, emission at 647 nm). The panels (c, c', c''), (f, f', f'') and (i, i', i'') show the overlay images of corresponding staining of FPNPs and To-PRO^TM^-3 along with DIC images. Yellow arrows (in d, e, f, g, h and i) indicate the hair cells stained with To-PRO-3^TM^. White arrows (in d', d'', e', e'', f', f'', g', g'', h', h'' and i', i'') indicate the defective hair cells by incubation with 1,4-BDA/FPNPs (B) or 1,8-ODA/FPNPs (C). The enlarged images of the white boxed areas in (a, a', a'', b, b', b'' and c, c', c'') are shown in (d, d', d'', e, e', e'' and f, f', f''), respectively. Scale bars 50 μm (f, f', f''), 100 μm (c, c', c'', i, i', i'').

Figure S9 show FL emission spectra of FPNPs prepared with different aliphatic diamines (1,4-butanediamine; 1,4-BDA, 1,8-octanediamine; 1,8-ODA, 1,12-dodecanediamine; 1,12-DDA) used as passivation agents. When 1,12-ODA was used, fluorescence was not observed (Figure S9d). TEM image of FPNPs prepared from 1,12-DDA shown no change, as compared to as-preapred non-fluorescent PDA (Figure S10e).


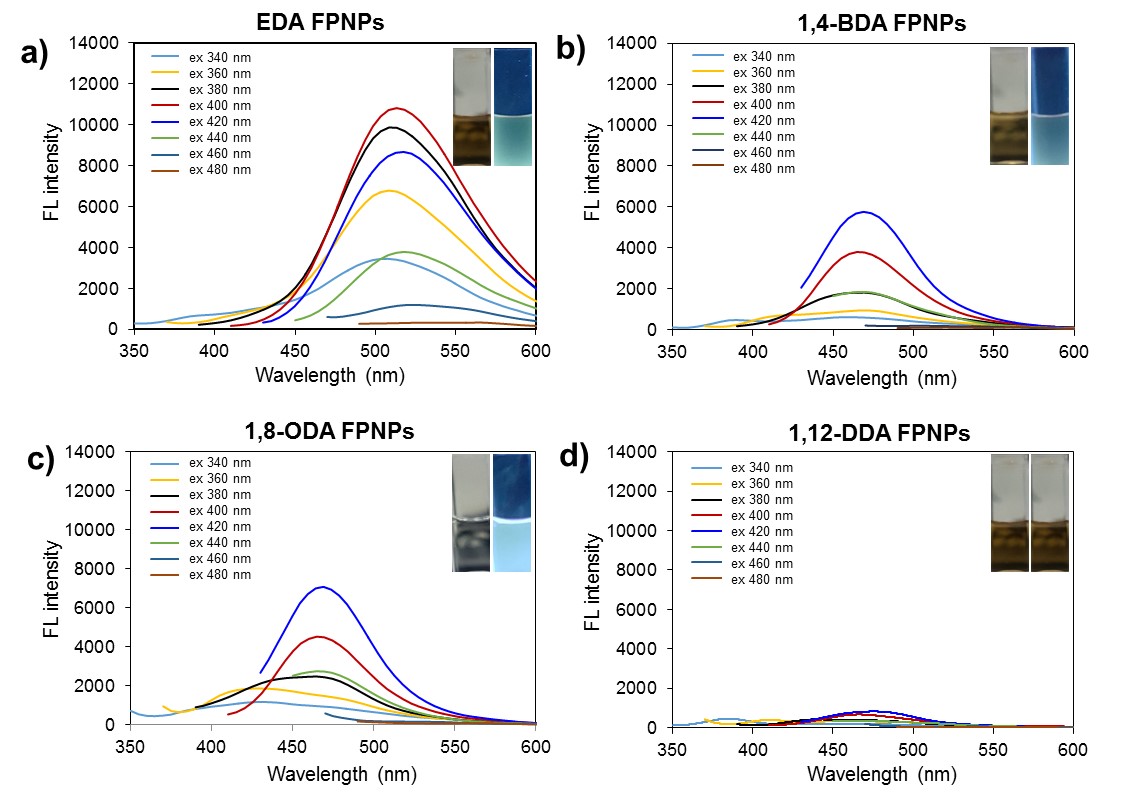


**Figure S9.** FL emission spectra the FPNPs prepared from (a) EDA, (b) 1,4-BDA, (c) 1,8-ODA and (d) 1,12-DDA used as passivation agents (50 μg mL^-1^).


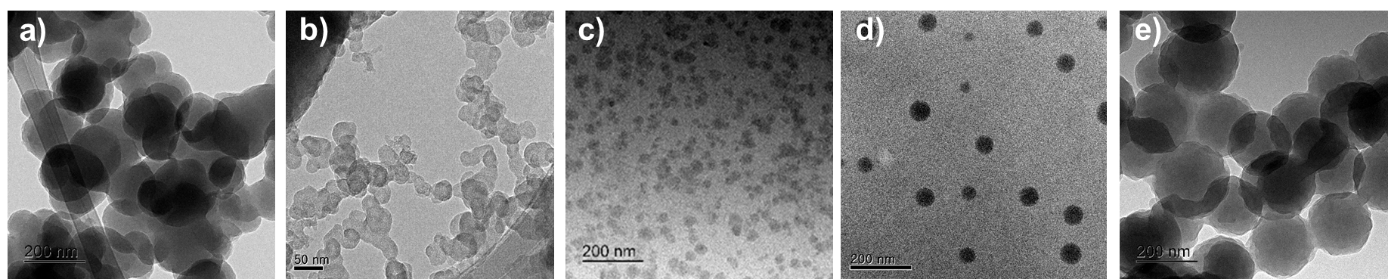


**Figure S10.** TEM images the FPNPs prepared from (b) EDA, (c) 1,4-BDA, (d) 1,8-ODA and (e) 1,12-DDA used as passivation agents. (a) is TEM image of PDA.

**
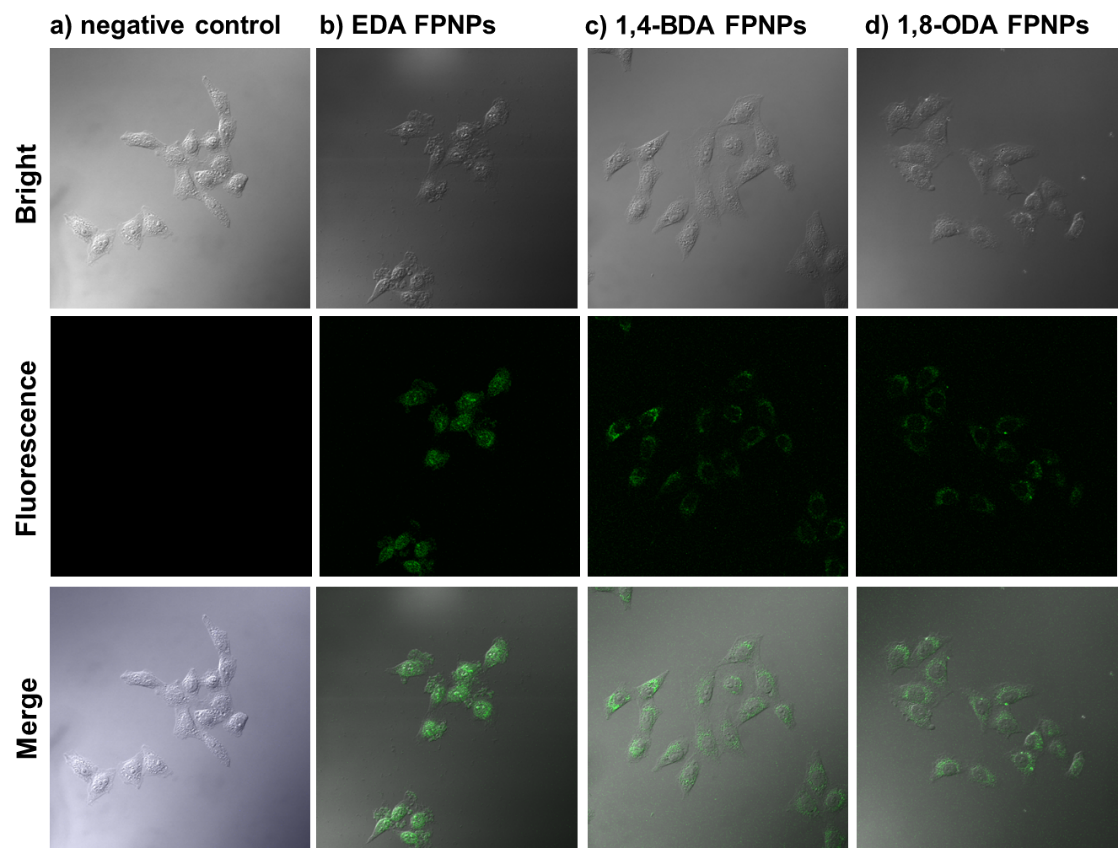
**

**Figure S11.** The cell permeability of FPNPs prepared from (b) EDA, (c) 1,4-BDA, (d) 1,8-ODA. (a) is negative control.
